# sTLR4/MD-2 complex inhibits colorectal cancer in vitro and in vivo by targeting LPS

**DOI:** 10.18632/oncotarget.10496

**Published:** 2016-07-08

**Authors:** Yan Zou, Fengxian Qin, Jifei Chen, Jie Meng, Liuhua Wei, Chunlin Wu, Qiaoyun Zhang, Dong Wei, Xiang Chen, Hao Wu, Xiaoli Chen, Shengming Dai

**Affiliations:** ^1^ Medical Science Laboratory, The Fourth Affiliated Hospital of Guangxi Medical University, Liuzhou, Guangxi, 545005, P.R. China

**Keywords:** sTLR4/MD-2 complex, CRC, LPS, pro-inflammatory cytokine, migration cytokine

## Abstract

Colorectal cancer (CRC) is aggressive and associated with TLR4-MD-2 signaling. Toll-like receptor 4 (TLR4) and myeloid differentiation protein 2 (MD-2) were highly expressed in human CRC. The soluble form of extracellular TLR4 domain (sTLR4) and MD-2 may have important roles in binding lipopolysaccharide (LPS). In this study, sTLR4 and MD-2 protein and prepared sTLR4/MD-2 complex were synthesized successfully to restrain LPS-TLR4/MD-2 activation by competing with cellular membrane TLR4 for binding LPS. The sTLR4/MD-2 complex can significantly attenuate LPS induced pro-inflammatory and migration cytokine production in vitro and in vivo, and inhibit the effect of LPS on the cell cycle, migration and invasion of human CRC cells in vitro. Administration of sTLR4/MD-2 complex protected mice from tumor both in xenograft and implantation metastasis model. The sTLR4/MD-2 complex treated mice had smaller tumor, less body weight loss and lower expression of inflammatory cytokines. Here, the azoxymethane/dextran sulfate sodium salt (AOM/DSS) murine model was used as an experimental platform to simulate the physiological and pathological processes of cancers associated with chronic intestinal inflammation. AOM/DSS-induced tumors were inhibited in mice treated by sTLR4/MD-2 complex. It is demonstrated in our study that sTLR4/MD-2 complex could inhibit CRC by competing with binding LPS, raising the complex's possibility of a new prevention agent against CRC.

## INTRODUCTION

Colorectal cancer (CRC) is the fifth most common cancer and the third biggest cause of neoplasm-related deaths in digestive system across China [1]. Despite considerable investments and remarkable advances in the management of cancer, the overall survival (OS) for this disease has changed little over the past 20 years. CRC is mainly caused by active ulcerative colitis (UC) or Crohn's disease (CD) with impact of chronic inflammation on its development. It is well known that chronic infection and inflammation are considered as two major contributors to tumorigenesis and tumor progression [2]. Chronic inflammation and the increased turnover of epithelial cells lead to the development of low- and high-grade dysplasia which may further transform into CRC.

Toll-like receptors (TLRs) signaling plays a vital role in cancers such as ovarian, pancreatic, lung, liver, gastric and colon cancer and serves as a major contributor to chronic inflammation at the same time [3–7]. TLRs recognize pathogen-associated molecular patterns (PAMPs) and activate downstream transcription factors to produce various pro-inflammatory cytokines and clear invading pathogens [8]. However, excessive inflammatory responses initiated by TLRs could disrupt immune homeostasis and result in immunopathological conditions [9]. Among TLRs, Toll-like receptor 4 (TLR4) was discovered as a sensing receptor for bacterial lipopolysaccharide (LPS) [10]. Membrane bound TLR4 recognizes LPS and signals with enhanced efficiency after forming a receptor complex with accessory proteins including myeloid differentiation protein 2 (MD-2), LPS binding protein, and CD14 [11–13]. Docking the LPS-CD14 complex onto the TLR4/MD-2 complex initiates signaling through both the myeloid differentiation primary response 88 (MyD88) and Toll/IL-1 receptor-domain-containing adapter-inducing interferon-β (TRIF) pathways [14]. MyD88-dependent signaling activates nuclear factor-κB (NF-κB) and leads to the production of pro-inflammatory cytokines such as IL-6, tumor necrosis factor α (TNF-α) and IL-12. Alternatively, TLR4 signaling can activate the TRIF pathway that acts through interferon (IFN) regulatory factor 3 to promote the production of type I IFN (IFN α/β), IFN-inducible gene products and an immune regulatory response [15]. However, excessive inflammatory responses triggered by TLRs can disrupt immune homeostasis. High TLR4 expression, found in a variety of tumors including CRC [16], intensely activates the related signaling pathways, promotes the secretion of inflammatory cytokines and accelerates disease progression. Contemporary studies highlighted a key function of the TLR system in the development of colitis-associated tumor, suggesting TLR4's role in CRC development and progression and its function as a potential prognostic marker of CRC [4, 17, 18].

In light of the crucial role of TLR4 in the development of CRC, inhibition of LPS-induced TLR4 signaling may be valuable for the therapeutic prevention from CRC. Since LPS responses are dependent on dimerization of TLR4/MD-2 instead of TLR4 or MD-2 alone, various methods were used to restrain the activity of TLR4/MD-2. Four-hydroxy-2-nonenal, the lipid peroxidation products, is used to suppress TLR4 activation by blocking TLR4 dimerization [19]. Eritoran (also known as E5564), second-generation lipid A analog, competes with LPS for the same hydrophobic binding pocket of MD-2 and induces a different conformational change to reduce the stability of TLR4/MD-2 complex and inhibits TLR4 signaling [20, 21]. But it did not reduce 28-day mortality in patients with severe sepsis when compared with placebo [22, 23]. Therefore, new effective antagonists are urgently needed to be discovered.

In order to find a new prevention agent, a soluble form of extracellular TLR4 domain (sTLR4) and MD-2 is prepared to form a sTLR4/MD-2 complex to inhibit TLR4 signaling. This complex could inhibit the binding of LPS to TLR4 on cell surface and down-regulate LPS-induced inflammation in vitro and in vivo. It suppressed the invasion of human's CRC cells and tumor generation in vitro whilst restrained tumor development effectively in mouse model in vivo. In summary, sTLR4/MD-2 complex could inhibit CRC by competing with binding LPS to raise its possibility of a new prevention agent against CRC.

## RESULTS

### TLR4 and MD-2 expression and its association with the clinic pathological characteristics of CRC patients

**Figure 1:**
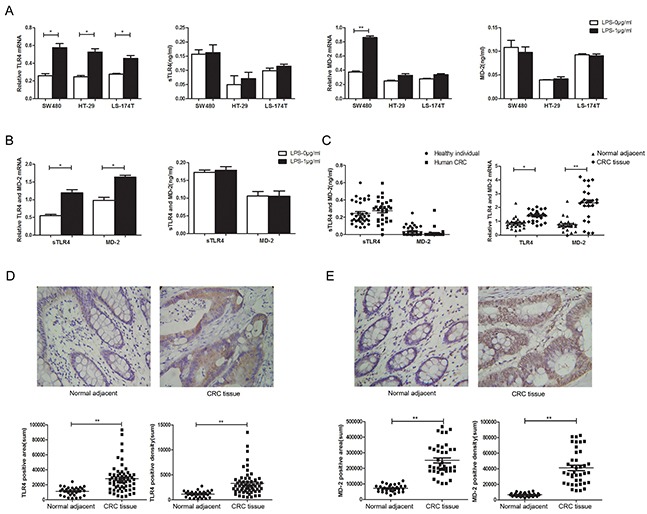
TLR4 and MD-2 expression and its association with the clinic pathological characteristics of CRC patients Expression of TLR4 and MD-2 were analyzed by RT-PCR and ELISA in CRC cells **A.** and PMA-induced THP-1 cell **B.** Expression of TLR4 and MD-2 were analyzed by ELISA (left) and RT-PCR (right) in CRC patients **C.** Immunohistochemistry for TLR4 **D.** and MD-2 **E.** in CRC patients from surgical samples of controls (normal adjacent tissue, n = 10) and patient samples (CRC tissue, n = 33) (only 1 picture per group was shown). Quantification of positive area (D and E, bottom, left) and positive density (D and E, bottom, right) were both shown. *P < 0.05, **P < 0.01, data were analyzed by unpaired Student's t test. ×400 magnification

Macrophages are one of the major sources of pro-inflammatory cytokines involved in inflammatory diseases and inflammation-related cancers. To identify the effect of LPS treatment on TLR4-signaling, we examined the expression of TLR4 and MD-2 both in macrophages and CRC cells. Although RT-PCR assay revealed LPS treatment enhanced TLR4 and MD-2 expression both in macrophages and CRC cells at the mRNA levels in consistence with previous studies [26, 27], no statistical significance was found between LPS treatment and PBS treatment at protein levels in cell culture supernatant (Figure 1A). The same went for PMA-induced THP-1 cells (Figure 1B). Importantly, the physiological levels of the serum sTLR4 and MD-2 concentration were also confirmed in CRC patients (Figure 1C, left). The serum sTLR4 and MD-2 concentrations had no statistical significance between CRC patients and healthy individuals. But RT-PCR assay revealed high expression of TLR4 and MD-2 in CRC tissue samples (Figure 1C, right). Meanwhile, the clinic pathology showed high expression of TLR4 and MD-2 by immunohistochemistry (Figure 1D, 1E). The expression of TLR4 and MD-2 was considerably increased in CRC cells and THP-1 cells after LPS-treatment, which was consistent with that in CRC patients.

### Successful preparation of sTLR4 and MD-2 protein

sTLR4 and MD-2 protein were purified using Ni^2+^ affinity chromatography and HiPrep Sephacryl HR columns. The fidelity of gene and protein was confirmed by PCR, DNA sequencing, SDS-PAGE (Coomassie staining) and Western blotting (data not shown). We chose corresponding optimal concentration of sTLR4, MD-2 and sTLR4/MD-2 complex by dose dependent manner.

### sTLR4/MD-2 complex restrains the binding of LPS to cellular membrane TLR4

**Figure 2:**
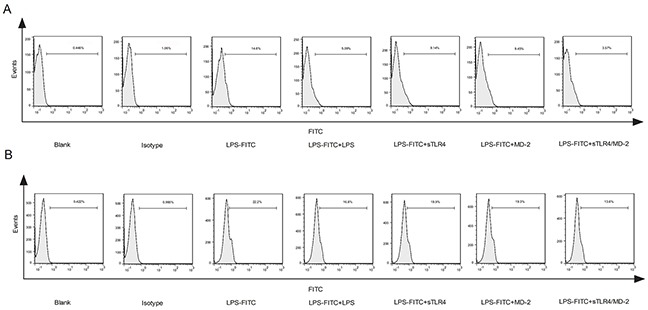
sTLR4/MD-2 complex restrains the binding of LPS to cellular membrane TLR4 **A.** PMA-induced THP-1 cells or **B.** SW480 cells were treated with PBS, Isotype (FITC-IgG), LPS-FITC (1.0 μg/ml), LPS + LPS-FITC (both 1.0 μg/ml), LPS-FITC + sTLR4 (LPS-FITC 1.0 μg/ml + sTLR4 5.0 μg/ml), LPS-FITC + MD-2 (LPS-FITC 1.0 μg/ml + MD-2 1.25 μg/ml) or LPS-FITC + sTLR4/MD-2 (LPS-FITC 1.0 μg/ml + sTLR4/MD-2 6.25 μg/ml), respectively. The fluorescence intensity of cells in each group was determined by flow cytometry. Representative images of three independent experiments were shown.

To evaluate the effect of sTLR4/MD-2 complex on competing cellular membrane TLR4 for binding to LPS, sTLR4, MD-2 or sTLR4/MD-2 complex was respectively added to PMA-induced THP-1 cells. The cell surface binding of LPS-FITC was examined. As shown in Figure 2A, the fluorescence intensity of sTLR4/MD-2 complex treated cells was significantly reduced, whereas sTLR4 or MD-2 alone could not inhibit the fluorescence intensity as effectively as sTLR4/MD-2 complex. There was a similar result when sTLR4/MD-2 complex was added to SW480 cells (Figure 2B). These results clearly demonstrated that sTLR4/MD-2 complex could compete with cellular membrane TLR4 for binding to LPS.

### sTLR4/MD-2 complex inhibits cell cycle, migration and invasion of SW480 cells by targeting LPS

**Figure 3:**
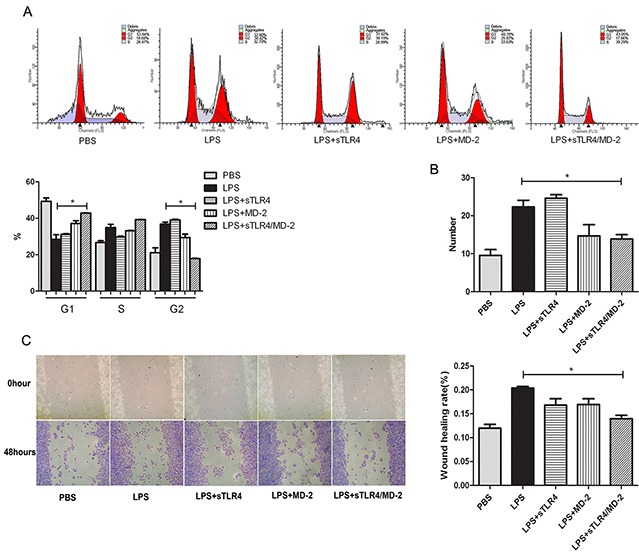
sTLR4/MD-2 complex inhibits the cell cycle, migration and invasion of CRC cells induced by LPS SW480 cells were incubated with PBS, LPS (1.0 μg/ml), sTLR4 (LPS 1.0 μg/ml + sTLR4 5.0 μg/ml), MD-2 (LPS 1.0 μg/ml + MD-2 1.25 μg/ml), sTLR4/MD-2 (LPS 1.0 μg/ml + sTLR4/MD-2 6.25 μg/ml) for 24 hours (the cell cycle and invasion assay) or 48 hours (the cell migration assay), respectively. **A.** Cell cycle analysis (upper), quantitative results (bottom). **B.** Transwell Matrigel invasion assay of SW480 cells was analyzed. ×100 magnification. **C.** Wound migration assay of SW480 cells (left) and quantitative results (right). ×400 magnification. *P < 0.05, data were analyzed by One-way ANOVA test with Graphpad Prism 5. Representative images of three independent experiments were shown.

To evaluate the effect of sTLR4/MD-2 complex on cell cycle of SW480 cells induced by LPS, the flow cytometry was performed after the cells were incubated with various proteins. The results showed that there was significant difference in cell cycle distribution in sTLR4/MD-2 complex group (Figure 3A). Then, we evaluated the effect of sTLR4/MD-2 complex on migration and invasion of CRC cells by wound migration and transwell matrigel invasion assays. sTLR4/MD-2 complex significantly inhibited SW480 cells motility induced by LPS. The wound size in sTLR4/MD-2 complex group was wider than that in LPS group (Figure 3C). Same as the results of the wound healing assay, the invasion ability of SW480 cells was significantly suppressed when treated with sTLR4/MD-2 complex (Figure 3B). In addition, our results found that sTLR4 or MD-2 alone could not inhibit the cell cycle, migration and invasion of SW480 cells as effectively as sTLR4/MD-2 complex.

### sTLR4/MD-2 complex inhibits the expression of pro-inflammatory cytokine and migration cytokine through NF-κB signaling in SW480 cells induced by LPS

**Figure 4:**
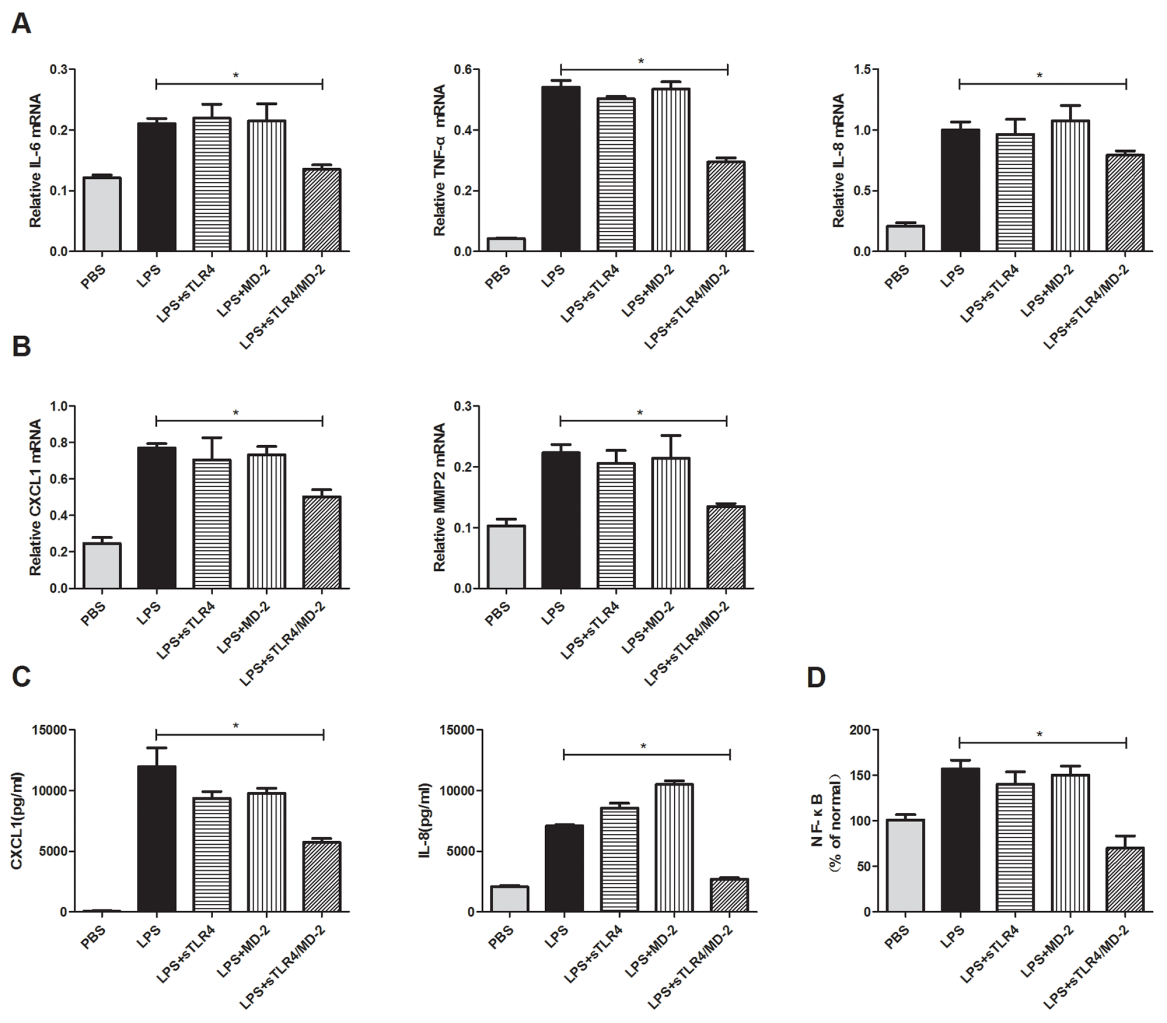
sTLR4/MD-2 complex inhibits the expression of pro-inflammatory cytokine and migration cytokine in SW480 cells induced by LPS Quantitative RT-PCR analyses of genes levels were performed on inflammatory cytokines IL-6, TNF-α and IL-8 **A.** migration related cytokines CXCL1 and MMP2 **B.** ELISA analyses of protein levels were performed on inflammatory and migration related cytokines IL-8 and CXCL1 **C.** of SW480 cells treated with PBS, LPS (1.0 μg/ml), sTLR4 (LPS 1.0 μg/ml + sTLR4 5.0 μg/ml), MD-2 (LPS 1.0 μg/ml + MD-2 1.25 μg/ml), sTLR4/MD-2 (LPS 1.0 μg/ml + sTLR4/MD-2 6.25 μg/ml), respectively. Data were expressed as related mRNA change over unchallenged SW480 cells group with error bars denoting standard error of the mean. **D.** Effect of sTLR4/MD-2 complex on LPS-induced NF-κB activity in SW480 by ELISA. NF-κB quantitative analysis is shown. Values were means ± SD of 3 measurements. *P < 0.05, and data were analyzed by One-way ANOVA test with Graphpad Prism 5.

To explore sTLR4/MD-2 complex's role in protection, SW480 cells were incubated with LPS plus corresponding protein. Then the inflammatory cytokines and migration related molecules were detected. The production of LPS-induced genes (TNF-α, IL-8 and IL-6) and the migration related genes (CXCL1 and MMP2) were suppressed after being treated with sTLR4/MD-2 complex (Figure 4A, 4B). But sTLR4/MD-2 complex only significantly decreased expression of CXCL1 and IL-8 in SW480 cells supernatant at protein levels rather than that of TNF-α, IL-6 and MMP2 (Figure 4C). In addition, our results found that sTLR4 or MD-2 alone could not inhibit the pro-inflammatory cytokine and migration cytokine in SW480 cells induced by LPS as effectively as sTLR4/MD-2 complex. Since p65 is the major component of NF-κB in LPS stimulated cancer cells, we determined the p65 activity of nuclear extracts by ELISA (Figure 4D). In the presence of sTLR4/MD-2 (LPS 1.0 μg/ml + sTLR4/MD-2 6.25 μg/ml), NF-κB activity was significantly suppressed.

### sTLR4/MD-2 complex protects mice from tumor

**Figure 5:**
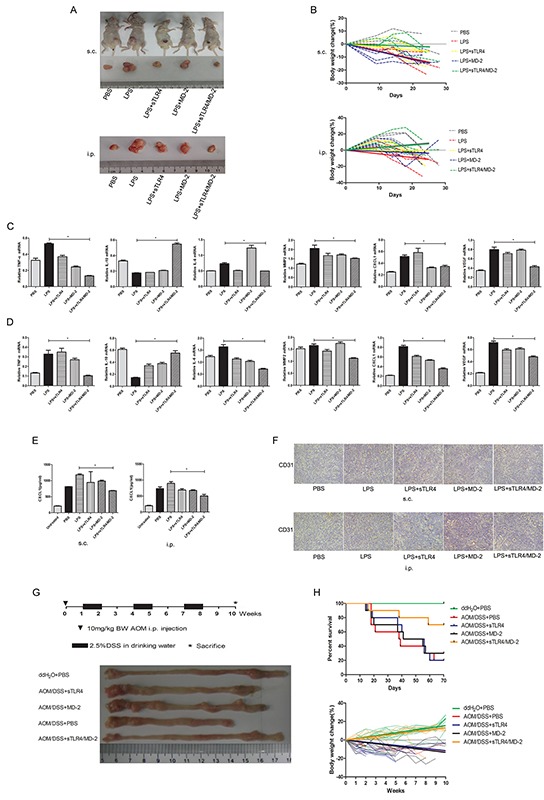
sTLR4/MD-2 complex protects mice from tumor In the xenograft model and implantation metastasis model, nude mice were injected s.c. with SW480 cells (2×10^6^/mouse) in 100 μl PBS in the flank or i.p. with the same cells (4 mice per group). For treatment, 5 days after tumor cells inoculation, mice were administered with different doses of PBS, LPS (LPS 1.5 g/kg), sTLR4 (LPS 1.5 g/kg + sTLR4 0.2 g/kg), MD-2 (LPS 1.5 g/kg + MD-2 0.05 g/kg), sTLR4/MD-2 (LPS 1.5 g/kg + sTLR4/MD-2 0.25 g/kg) and then repeated three times on days 10, 15 and 20. **A.** The tumor volumes of different group mouse. s.c. (upper) and i.p. (bottom). **B.** Body weights of different group mouse. s.c. (upper) and i.p. (bottom). The trend line fitted with linear regression. Quantitative RT-PCR analysis of genes levels for inflammatory cytokines (IL-6, TNF-α and IL-10), migration related cytokines (CXCL1 and MMP2) and VEGF. s.c. **C.** and i.p. **D, E.** ELISA analysis of protein level for inflammatory and migration related cytokines CXCL1. s.c. (left) and i.p. (right). **F.** Immunohistochemical staining of CD31 expression. s.c. (upper) and i.p. (bottom). In AOM/DSS-induced CRC model, mice were administered with a single intraperitoneal injection of the AOM (10 mg/kg body weight) and subsequently three rounds of 2.5% DSS orally (7 days each round and 14 days interval between the 2 rounds) (10 mice per group). For treatment, 7 days after injection of the AOM, mice were administered with different doses of PBS, sTLR4 (0.2 g/ kg), MD-2 (0.05 g/kg), sTLR4/MD-2 (0.25 g/kg) by i.p., and then repeated every 5 days until the last week. **G.** Schematic experimental procedure for groups treated with AOM and DSS (upper), macroscopic observation of the normal adjacent regions of colons from each groups (bottom) at the end of the 10th week (only 1 animal per group was shown). **H.** Survival curve (upper) and body weights (bottom) of different group mouse induced by AOM/DSS. *P < 0.05, data were analyzed by One-way ANOVA test with Graphpad Prism 5. ×200 magnification

We tried to examine the prevention effect of sTLR4/MD-2 complex in mice tumor model. Nude mice xenograft model and peritoneal implantation metastasis model were used to study the effect of sTLR4/MD-2 complex under LPS treatment. As expected, administration of sTLR4/MD-2 complex after LPS injection obviously had smaller tumor volume in both two models compared with LPS treated group (Figure 5A). No tumor metastasis was found in viscera including liver, spleen and kidney (data not shown). Compared with sTLR4/MD-2 complex treated mice, the LPS treated mice had more body weight loss (Figure 5B). In addition, the mRNA level of inflammatory cytokines was detected by PCR in tumor tissue. Lower expression of inflammatory cytokines (TNF-α and IL-6) were shown in mice treated by sTLR4/MD-2 complex than that in LPS treated mice, whereas IL-10 expression was increased both in s.c. (Figure 5C) and i.p. group (Figure 5D). There was a similar result when the inflammatory cytokines in serum were examined by ELISA (Figure 5E). At the same time, compared with mice treated by sTLR4/MD-2 complex, the LPS treated mice had high expression of microvascular formation factor VEGF at the mRNA level (Figure 5C, 5D). The CD31-positive cells in small and intermediate-sized vessels were shown as microvessel density for evaluation of angiogenesis and reendothelialization. In contrast, there is a low expression of CD31 after LPS injection followed by injecting sTLR4/MD-2 complex (Figure 5F). Finally, we used AOM/DSS-induced tumor model to investigate the effect of sTLR4/MD-2 complex on AOM/DSS-induced inflammation and tumor. We found sTLR4/MD-2 complex also had the inhibitory effect on colon length shortening and body weight loss to enhance survival rates (Figure 5G, 5H). In addition, our results showed that sTLR4 or MD-2 alone could not play a protective role as effectively as sTLR4/MD-2 complex.

## DISCUSSION

CRC is the third most common cancer in men and women. In addition, CRC becomes the third leading cause of cancer-related death with increasing incidence [28]. TLR4 has been detected in cell lines of many human cancers, including gastric, breast, lung, prostate and colon cancer [29]. Wang et al found that the high expression of TLR4 was significantly correlated with liver metastasis and associated with lower OS in colorectal cancer [30]. Bacterial infection stimulates the TLR/MYD88 pathway in tumor tissues, which is essential to the development and maintenance of an inflammatory microenvironment in gastrointestinal tumors [31]. TLR4 is overexpressed in mouse and human inflammation-associated CRC, and TLR4-deficient mice are strongly protected against colon carcinogenesis, suggesting that TLR4 expression on tumor cells promotes tumor progression directly or indirectly [32]. Therefore, targeting on TLR signaling may be a potential strategy to abrogate this inflammation-mediated effect in tumor progression [33, 34]. In our study, it is proved that TLR4 and MD-2 are significantly increased in LPS induced macrophages, CRC cells and tissues of CRC patients (Figure 1) as Nihon-Yanagi Y, et al reported [26, 27]. But whether and how sTLR4 sheds from cell surface is not clear. So we customized two ELISA kits of sTLR4 and MD-2 to detect their protein levels in CRC cell supernatant and the serum of CRC patients. But protein levels in cell culture supernatant and CRC patient's serum had no statistical significance. So it was postulated that sTLR4 and MD-2 are too low or our ELISA kit cannot detect the two proteins. We plan to custom a ELISA kit with higher sensitivity to perfect our experiment. As LPS was a major stimulator of TLR4 and could bind to TLR4 under the assistant of MD-2 [24], it was hypothesized that sTLR4 and MD-2 might have important roles in binding LPS. Thereupon we synthesized endotoxin-free sTLR4 and MD-2 protein, prepared sTLR4/MD-2 complex (1:1) and chose the optimal concentration by dose dependent manner for subsequent experiments on cells and animals (data not shown). It was confirmed in our results that only sTLR4/MD-2 complex could bind to LPS effectively but sTLR4 or MD-2 alone could not.

An important finding is that sTLR4/MD-2 complex restrained the binding of LPS to cell surface TLR4. Since LPS-TLR4 signaling plays crucial roles in the development of CRC [35], the effect of sTLR4/MD-2 complex on the biological characteristics of CRC cells was investigated. We examined the effect of LPS on CRC cell proliferation and apoptosis, which had no significant difference among each group (data not shown). Next, the cell cycle was tested, and it was significantly suppressed in sTLR4/MD-2 complex group (Figure 3A). In addition, cell migration and invasion were notably inhibited (Figure 3B, 3C). LPS-TLR4-MD-2 signaling leads to the activation of multiple signaling components and the production of pro-inflammatory cytokines subsequently [36]. In our study, the expression of inflammatory factors in SW480 cells was also evaluated (Figure 4). RT-PCR assay revealed a substantial decrease of multiple pro-inflammatory and migration cytokines in sTLR4/MD-2 complex treated cells, including TNF-α, IL-8, IL-6, CXCL1 and MMP2. The results of ELISA assay verified the decrease of IL-8 and CXCL1 in CRC cells treated with sTLR4/MD-2 complex (Figure 4C). These results of pro-inflammatory and migration cytokines were consistent with previous studies [37, 38]. Since these pro-inflammatory and migration cytokines constituted tumor microenvironment of CRC to affect tumor progression [18], it was suggested in our study that sTLR4/MD-2 complex can inhibit tumor progress (Figure 5A) whereas sTLR4 or MD-2 alone could not inhibit tumor progress as effectively as sTLR4/MD-2 complex. When CRC cells were treated with LPS and sTLR4/MD-2 complex for 24 hours, the p65 increases induced by LPS were significantly inhibited. The results clearly indicate that sTLR4/MD-2 complex inhibited LPS binding to TLR4 on the CRC cell membrane and suppressed NF-κB p65 translocation to the nucleus. Similar to the findings of the current study, LPS increased the migration and the invasion abilities of CRC cells by promoting NF-κB activation. Our study verified these results again.

Another important finding is that sTLR4/MD-2 complex bound to LPS to protect mice from tumor. Therefore, it would be intriguing to examine the function of sTLR4/MD-2 complex in vivo. The nude mice xenograft model and peritoneal implantation metastasis model were successfully constructed. Interestingly, it is found that the LPS, LPS+sTLR4 and LPS+MD-2 groups were more sensitive to CRC cells whereas sTLR4/MD-2 complex group had smaller tumors. Just as is reported in the previous study, silencing TLR4 signaling in tumor cells resulted in reduced tumor formation [39]. Because TLR4/MD-2 antibody therapy had been applied to treat lymphoedema by targeting lymphatic vessels in mouse models [40], our study proposed an unrecognized role of sTLR4/MD-2 complex in CRC prevention. Furthermore, there were less body weight loss and lower expression of inflammatory cytokines in sTLR4/MD-2 complex treated group compared with the LPS group, sTLR4 and MD-2 groups respectively. Interestingly, an increase of IL-10 expression was observed in sTLR4/MD-2 complex treated group. High expression of IL-10, an anti-inflammatory cytokine, can inhibit the inflammation. Our study revealed that sTLR4/MD-2 complex could prevent the progression of tumor induced by LPS. VEGF and CD31 expression levels are significantly correlated with human CRC TNM stage, histological grade, tumor size and metastasis. Furthermore, concomitant expression of VEGF and CD31 has been shown to be associated with increased potential for carcinoma growth and metastasis in human gastric cancer [41]. Besides, our study also revealed that sTLR4/MD-2 complex could inhibit the overexpression of VEGF and CD31. The AOM/DSS model employed in this study (also called as “two step model”, i.e. one injection of AOM and three cycles of DSS) is a chronic inflammation-related model in which the initial inflammatory microenvironment is essential to promote and accelerate the malignant progression which starts after the AOM administration [42]. The damage of inflammatory microenvironment can activate the TLRs signal. So the sTLR4/MD-2 complex was used to intervene in AOM/DSS-induced inflammation and tumor, and an inhibitory effect was found. Above all, the results revealed sTLR4/MD-2 complex had an inhibitory effect on inflammatory stimulation both induced by LPS and in other forms when activating TLR4 signal pathway. Finally, sTLR4/MD-2 complex could effectively inhibit the progress of the tumor.

In conclusion, LPS-induced TLR4 signaling in CRC affects tumor growth, adhesiveness and metastatic capability. The blockage of this signaling pathway may prove to be a novel method in controlling the development and progression of cancer. Here, we successfully prepared endotoxin-free sTLR4/MD-2 complex which competed with cellular membrane TLR4 by targeting LPS and then suppressed human CRC in vitro and in vivo, raising the possibility of a new prevention agent against CRC. Further studies need to be investigated in preclinical and clinical trials of the prevention and therapeutic agent used.

## MATERIALS AND METHODS

### Cell culture

The human CRC cell lines SW480, LS174T, HT-29 and macrophage-like cell THP-1 were obtained from Shanghai Institutes for Biological Sciences. The cells were cultured in DMEM (Gibco) or RPMI 1640 medium (Gibco) with 10% fetal bovine serum (FBS) (Gibco), 100 U/ml penicillin G, 100 μg/ml streptomycin and then incubated at 37°C in a humidified atmosphere containing 5% CO_2_ and 95% air. To induce the THP-1 cells to macrophages, the cells were cultured with 100 ng/ml Phorbol-12-myristate-13-acetate (PMA, Sigma-Aldrich) in RPMI 1640 containing 10% FBS for 24 hours and further incubated in the medium absence of PMA for another 24 hours.

### Preparation of extracellular TLR4 domain and MD-2

A soluble form of extracellular TLR4 domain (sTLR4) consists of the putative extracellular domain (Met^1^-Lys^631^) and a 6×His tag at the C-terminalend. sTLR4 cDNA was constructed by PCR. The sense primer and antisense primer used were 5′-CCCAAGCTTGCCACCATGATGTCTGCCTCGCGCCTGG-3′ and 5′-CGCGGATCCTTAGTGATGGTGATGGTGATGCTTATTCATCTGACAGGTGATATTC-3′, respectively. Constructed sTLR4 cDNA was confirmed by DNA sequencing. sTLR4 cDNA was subcloned into pTT5 plasmid vector using HindIII and BamHI site. sTLR4 protein was expressed by CHO cell expression system and then purified using Ni^2+^ affinity chromatography and HiPrep Sephacryl HR columns (GE Healthcare).

MD-2 cDNA was constructed by PCR. The sense primer and antisense primer used were 5′- CCCATATGATGGAAGCGCAGAAACAGTACTGGG-3′ and 5′- CCCTCGAGTAAGTTAGAGTTCGGCTGGTGCAGGATA-3′, respectively. The amplified 435 bp PCR products were purified and digested with NdeI and XhoI (TaKaRa) and then ligated into pET28α (+) (Novagen) at 16°C overnight. The ligation products were transformed into JM109, positive transformants with the appropriate insert were screened on medium supplemented with 50 μg/ml kanamycin, and the recombinant plasmids were identified by PCR amplification with the MD-2 sense and MD-2 antisense primers and DNA sequencing. Then protein expression was induced with 0.2 mM isopropyl-1-thio-β-D-galactopyranoside (IPTG) for 7 hours. The cells were centrifuged (5,000 rpm for 10 minutes) at 4°C and resuspended in 50 mM phosphate buffer (pH 7.4). The cells were lysed by sonication, and the cellular debris was removed by centrifugation at 8000 rpm for 15 minutes at 4°C. Purification of the MD-2 recombinant protein was performed by Ni-NTA (Nitrilotriacetic acid) His-bind resin as described by the manufacturer (GE Healthcare) and HiPrep Sephacryl HR columns.

To remove endotoxin, the protein solutions were treated with Detoxi-Gel Endotoxin Removing Gel (Thermo). The purity of sTLR4 and sMD-2 proteins was over 95% as confirmed by silver stained sodium dodecyl sulfate–polyacrylamide gel electrophoresis (SDS-PAGE). LPS contamination was under 0.1 EU/μg protein as determined by the Limulus amebocyte lysate assay (BioWhittaker). sTLR4/MD-2 complex was prepared according to the molar ratio of 1: 1 [24].

### Wound migration assay

The SW480 cells were grown in six-well plates to a confluent monolayer and subsequently wounded with sterile pipette tips. The wounded monolayers were then incubated with LPS (Sigma-Aldrich) and corresponding protein as indicated in Figure 3 for 48 hours. The wound area was measured under microscope. The percentage of wound healing rate was estimated as follows: Wound healing rate % = [1-(wound width at 48 hours/wound width at 0 hour)] × 100%. ×100 microscopic fields under microscope.

### Transwell Matrigel invasion assay

Tumor cell invasion was performed using Transwell system (Millipore) with 8 μm-pore polycarbonate filter membrane. The upper chamber was covered with Matrigel (Sigma-Aldrich) and incubated at 4°C overnight. The upper chamber was then seeded with 1×10^4^ SW480 cells incubated with LPS and corresponding protein as indicated in Figure 3 and inserted into the lower chamber containing complete medium. After incubation at 37°C in 5% CO_2_ for 24 hours, the cells on the interior of upper chamber were removed, and the polycarbonate membranes were stained with 0.1% crystal violet (BASO) for 10 minutes. The number of migrating cells was counted in eight randomly selected ×400 microscopic fields under microscope.

### Binding ability of sTLR4/MD-2 complex to LPS

The binding ability of sTLR4/MD-2 complex to LPS was examined by flow cytometry. LPS-FITC (Sigma-Aldrich) was premixed with LPS or corresponding protein as indicated in Figure 2 and then incubated with SW480 or PMA-induced THP-1 cells for 1 hour at 37°C. The fluorescence intensity of cells was determined.

### Cell cycle analysis

To examine the effect of sTLR4/MD-2 complex on cell cycle, SW480 cells (1×10^6^) were washed by PBS for three times, and then fixed with 70% cold ethanol. Cell cycle analysis was carried out by flow cytometry after propidium iodide staining. Three independent experiments were performed on the cells in independent cultures at three different times.

### RT-PCR

RNAiso Plus reagent (TaKaRa) was used to extract total RNA from the culture cells and tissues. PrimeScript™ RT Reagent Kit (TaKaRa) with gDNA Eraser was incubated with total RNA for reverse transcription. Premix Taq™ (Ex Taq Version 2.0 plus dye, TaKaRa) was used in PCR reaction. The entire process was carried out in accordance with the manufacturer's instructions. The synthesized cDNA was stored at −80°C. Primers used to amplify the genes and the internal reference gene-glyceraldehyde-phosphate dehydrogenase (GAPDH) of mouse and human were listed in Table 1 and Table 2. The 25 μl PCR reaction included Premix Taq, 20 μM primers, cDNA and double-distilled water. PCR reaction without a template was used as the negative control. PCR reaction conditions were as follows: 35 cycles at 98°C for 10 seconds, 50-60°C for 45 seconds, and 72°C for 45 seconds. PCR products were separated on a 2% agarose gel with GoldView I staining (Solarbio). The results of gel image were analyzed by using the AlphaImager gel analysis system (Protein simple). Each analysis was repeated three times, and the mean was obtained in order to reduce error. The semiquantitative value was expressed as an integrated optical density ratio (riOD), where riOD = (average gene electrophoresis optical density × area)/ (average GAPDH electrophoresis optical density × area). A ratio of > 0.5 was considered as positive expression, and a ratio of ≤ 0.5 was considered as negative expression.

**Table 1:** Primer sequences used for RT-PCR in mouse

Primer	bp	Annealing temperature (°C)	Sequence (5′→3′)
GAPDH F	231	55	TGATGACATCAAGAA GGTGGTGAAG
GAPDH R			TCCTTGGAGGCCAT GTAGGCCAT
TNF-α F	223	59	GGCAGGTCTACTTTGGAGTCA
TNF-α R			CACTGTCCCAGCCATCTTGTG
IL-6 F	141	54	GTTCTCTGGGAAATCGTGGA
IL-6 R			GCATTGGAAATTGGGGTAGG
IL-10 F	263	48	CTCGTTTGTACCTCTCTCCG
IL-10 R			ATCTCCCTGGTTTCTCTTCC
VEGF F	316	55	GCTACTGCCGTCCGATTGA
VEGF R			CGCTTTCGTTTTTGACCCTT
CXCL1 F	268	55	ACCCGCTCGCTTCTCTGT
CXCL1 R			CACCTTTTAGCATCTTTTGG
MMP2 F	499	52	CGAGACCGCTATGTCCACT
MMP2 R			CACTGTCCGCCAAATAAAC

**Table 2:** Primer sequences used for RT-PCR in human

Primer	bp	Annealing temperature (°C)	Sequence (5′→3′)
GAPDH F	289	55	GCGAGATCCCTCCAAAATC
GAPDH R			CATGAGTCCTTCCACGATACC
IL-6 F	228	55	CTTCGGTCCAGTTGCCTTCT
IL-6 R			GCCTCTTTGCTGCTTTCACAC
IL-8 F	293	60	GACATACTCCAAACCTTTCCACC
IL-8 R			CAACCCTACAACAGACCCACAC
TNF-α F	719	60	GCCCCAATCCCTTTATTACCC
TNF-α R			GGCGATTACAGACACAACTCCC
TLR4 F	311	55	AGTTGAACGAATGGAATGTGC
TLR4 R			CTTCATGGATGATGTTGGCAG
MD-2 F	454	55	TGAAGCTCAGAAGCAGTATTGG
MD-2 R			GGTTGGTGTAGGATGACAAACTC
CXCL1 F	356	50	AGAACATCCAAAGTGTGAACG
CXCL1 R			GCTCAAACACATTAGGCACAA
MMP2 F	316	49	GCCCAAGAATAGATGCTGACT
MMP2 R			TCGGTAGGGACATGCTAAGTA

### Patients and human tissues

A total of 63 patients with CRC (27 females and 36 males) who underwent surgery between November 2014 and October 2015 at the Fourth Affiliated Hospital of Guangxi Medical University were investigated in this study. Information about patient demographics (age and sex) and tumor stage was obtained from clinical and pathological records (Table 3). Ethical approval for the project was obtained from the Fourth Affiliated Hospital of Guangxi Medical University Research Ethics Committee. For ELISA study, serum samples from 30 CRC patients and 35 healthy individuals were collected. For RT-PCR assay and immunohistochemical study, tissue samples from 10 normal adjacent tissues as well as 33 CRC tissues were used.

**Table 3:** Patient samples enrolled for ELISA and IHC-P detection

		Serum		Tissue	
		Healthy n (%)	CRC n (%)	Normal n (%)	CRC tissue n (%)
Gender	Female	17 (49)	13 (43)	4 (40)	14 (43)
	Male	18 (51)	17 (57)	6 (60)	19 (57)
Age	36-59	15 (43)	14 (47)	5 (50)	16 (48)
	60-69	10 (29)	10 (33)	3 (30)	12 (36)
	>69	10 (29)	6 (20)	2 (20)	5 (16)
Stage	I	-	4 (13)	-	5 (15)
	II	-	14 (47)	-	14 (43)
	III	-	7 (23)	-	9 (27)
	IV	-	5 (17)	-	5 (15)
Total		35	30	10	33

### Xenograft model and implantation metastasis model

Athymic nude mice (BALB/C, nu/nu) were from the Model Animal Research Center, Guangxi Medical University, China. All of the animals were maintained under pathogen-free conditions. The Animal Care and Ethics Committee at Guangxi Medical University approved all animal experiments in our study. Eight-week-old nude mice received subcutaneous injection (s.c.) in the flank with SW480 cells (2×10^6^/mouse) in 0.1 ml PBS as described in our previous study [25]. In addition, nude mice received intraperitoneal injection (i.p.) with the same cells to construct implantation metastasis model. Tumor growth and mortality was monitored every 2 days. For treatment, 5 days after tumor cell inoculation, mice were administered with sTLR4, MD-2, sTLR4/MD-2, LPS or PBS as indicated and then repeated three times on day 10, 15 and 20. The body weights were determined at different times.

### AOM/DSS-induced CRC model

Eight-week-old C57BL/6 mice were also from the Model Animal Research Center, Guangxi Medical University, China. Mice were administered with a single intraperitoneal injection of the AOM (Sigma-Aldrich, 10 mg/kg body weight) and subsequently three rounds of 2.5% DSS (Sigma-Aldrich) orally (7 days each round and 14 days interval between the 2 rounds) [42]. For treatment, 7 days after injection of the AOM, mice were administered with sTLR4, MD-2, sTLR4/MD-2, PBS by i.p., and then repeated every 5 days until the last week (Figure 5G, upper). At the end of the AOM/DSS protocol, sections of normal adjacent tissues and medial regions of tumors, and colon length were observed.

### Enzyme-linked immunosorbent assay (ELISA)

The protein levels of IL-8, IL-6, TNF-α (Ray Biotech), sTLR4, MD-2 (Ray Biotech, customed) and CXCL1 (R&D) were detected by using the specific ELISA kits according to the manufacturer's instructions. The minimum detectable dose of Human sTLR4 and MD-2 was determined to be 0.4 ng/ml and 1.6 ng/ml respectively. Three independent experiments were performed at three different times. NF-κB p65 content was evaluated by ELISA (Shanghai biorui). Briefly, 3 × 10^6^ SW480 cells per well were plated in 6-well dishes and incubated with PBS, LPS (1.0 μg/ml), sTLR4 (LPS 1.0 μg/ml + sTLR4 5.0 μg/ml), MD-2 (LPS 1.0 μg/ml + MD-2 1.25 μg/ml), sTLR4/MD-2 (LPS 1.0 μg/ml + sTLR4/MD-2 6.25 μg/ml) for 24 hours, respectively. Cell nuclear preparation was made by a nuclear extraction kit (Millipore).

### Immunohistochemical staining

Serial sections in thickness of 4μm were mounted on adhesion microscope slides (CITOGLAS). Sections were dewaxed in xylene and rehydrated in graded ethanol. Endogenous peroxidase activity was blocked by immersion in 0.3% hydrogen peroxide for 10 minutes. Immunoreactivity was enhanced by microwaving by incubating the tissue sections for 5 minutes in 0.1 M citrate buffer. Immunostaining was performed using anti-CD31 antibody (Abcam, ab28364), anti-TLR4 antibody (Abcam, ab13556), anti-MD-2 antibody (Abcam, ab24182), goat anti-rabbit antibody (ZSGB-BIO, PV-6001) was used as secondary antibody. The antigen-antibody reactions were visualized with the chromogen diaminobenzadine.

### Statistical analyses

Statistical testing was performed by unpaired Student's t test or One-way ANOVA test with Graphpad Prism 5 unless otherwise indicated. The trend line in body weight change was fitted with linear regression. Statistical significance was assumed at P < 0.05.
